# *MTHFR* and *F5* genetic variations have association with preeclampsia in Pakistani patients: a case control study

**DOI:** 10.1186/s12881-019-0905-9

**Published:** 2019-10-23

**Authors:** Feriha Fatima Khidri, Yar Muhammad Waryah, Faiza Kamran Ali, Hina Shaikh, Ikram Din Ujjan, Ali Muhammad Waryah

**Affiliations:** 1 Molecular Biology and Genetics Department, Medical Research Center, Liaquat University of Medical and Health Sciences, Jamshoro, Pakistan; 2 Department of Biochemistry, Liaquat University of Medical and Health Sciences, Jamshoro, Pakistan; 3 Department of Chemistry, Shaheed Benazir Bhutto University, Shaheed Benazir Abad, Pakistan; 4 Department of Gynaecology and Obstetrics, Liaquat University of Medical and Health Sciences, Jamshoro, Pakistan; 5 Department of Pathology, Liaquat University of Medical and Health Sciences, Jamshoro, Pakistan

**Keywords:** *F5*, *MTHFR*, Pakistan, Preeclampsia, Variants, *VEGFA*

## Abstract

**Background:**

To study the role of single nucleotide variants (SNVs) of genes related to preeclampsia in Pakistani pregnant women.

**Methods:**

After ethical approval and getting informed consent; 250 pregnant women were enrolled and equally divided into two groups (125 preeclamptic cases and 125 normotensive pregnant women). Demographic details and medical history were recorded, and 10 ml blood sample was obtained for DNA extraction. The tetra-primer amplification refractory mutation system (ARMS) assays were developed for assessing the variants of three preeclampsia related genes; *F5*, *MTHFR* and *VEGFA*. An association of six SNVs; *F5*:c.1601G > A (rs6025), *F5*:c.6665A > G (rs6027), *MTHFR*: c.665C > T (rs1801133), *MTHFR*: c.1286A > C (rs1801131), *VEGFA*: c.-2055A > C (rs699947) and *VEGFA*: c.*237C > T (rs3025039) with preeclampsia was determined by using different genetic models.

**Results:**

Genotyping of the SNVs revealed that patients with *MTHFR*:c.665C > T, have increased susceptibility to preeclampsia (CT versus CC/TT: OR = 2.79, 95% CI = 1.18–6.59; P* = 0.046 and CT/TT vs CC: OR = 2.91, 95% CI = 1.29–6.57; P* = 0.0497, in overdominant and dominant models, respectively), whereas *F5*:c.6665A > G, (A/G vs AA/GG: OR = 0.42, 95% CI = 0.21–0.84; P* = 0.038 in overdominant model) and *MTHFR*:c.1286A > C, (CC versus AA: OR = 0.36, 95% CI = 0.18–0.72; P* = 0.0392 in codominant model) have significantly decreased risk for preeclampsia. *F5*:c.1601G > A, *VEGFA: c.-2055A > C* and *VEGFA*: c.*237C > T variants revealed no relationship with the disease.

**Conclusion:**

This is the first case control study describing the protective role of *F5*:c.6665A > G against preeclampsia in any world population. In addition, the present study confirmed the association and role of *MTHFR* gene variations in the development of preeclampsia in Pakistani patients. Further genetic studies may be required to better understand the complex genetic mechanism of SNVs in preeclampsia related genes in pregnant women.

## Background

Preeclampsia has been estimated to affect 2–8% of pregnancies, causing 10–15% of maternal deaths worldwide [1, 2]. It is a multifactorial and complex disorder and various studies have proposed genetic, environmental, immunological and nutritional factors for its occurrence, though the exact cause largely remains debatable [3–5]. More than 70 candidate genes related to thrombophilia, blood pressure regulation, angiogenesis, hormones and lipid metabolism have been studied to detect an association with preeclampsia, however, results from these studies are inconsistent and conflicting [6].

*F5* (OMIM# 612309), *MTHFR* (OMIM# 607093) and *VEGFA* (OMIM# 192240) genes have been widely studied in association with preeclampsia. The products of these genes play a crucial role in the mechanism required for the normal development and functioning of the placenta [7, 8]. The functional genetic variations in the genes affect the thrombogenic and angiogenic properties which lead to abnormalities of the placenta and result in preeclampsia [9–11]. The common SNVs found in association with preeclampsia include *F5*:c.1601G > A (rs6025), *MTHFR*: c.665C > T (rs1801133), *MTHFR*: c.1286A > C (rs1801131), *VEGFA: c.-2055A > C* (rs699947) and *VEGFA*: c.*237C > T (rs3025039) [12–14]. The influence of the SNVs on disease outcome is variable among different world populations, possibly due to ethnic variations and the findings may not be generalized [15].

Pakistani population is genetically heterogeneous and have unique genetic profiles, several novel genes and alleles have been identified to help better understand the disease prediction and the course of pathogenicity. Limited literature is available for Pakistani patients explaining the interaction of genetic variations for prediction and understanding the pathogenic mechanisms of preeclampsia [16]. It is hypothesized that genotypes of SNVs of genes, crucial for development and the function of the placenta may reveal the novel genetic associations to predict the disease and its mode of manifestation. In the present study, 6 SNVs of *F5*, *MTHFR*, and *VEGFA* genes have been studied in patients with preeclampsia and normal controls. This study may help to better understand the genetics of preeclampsia and the role of variants in the related genes for better prognosis and management of the disorder.

## Methods

### Study design and participants

The proposed case-control study was conducted after approval from the Research Ethics Committee of Liaquat University of medical and health sciences (LUMHS), Jamshoro. Written informed consent was taken from all the participants. A total of 250 pregnant women (125 cases and 125 controls) were selected from labour room, wards and outpatient department of Gynaecology and Obstetrics units. It was attempted to recruit all preeclamptic patients admitted from March 2014 to Feb 2015. During this period 187 preeclamptic patients from 20 different districts of Sindh were admitted and after following exclusion criteria, 125 cases were recruited.

### Inclusion and exclusion criteria

Inclusion criteria for the preeclamptic woman was defined according to the American college of obstetrics and gynaecologists as the development of gestational hypertension (blood pressure ≥ 140/90 mmHg on two events at least 6 h apart) and significant proteinuria (≥ 0.3 g protein in 24-h urine specimen or ≥ 1+ on dipstick test) after 20 weeks of gestation in previously normotensive women. Severe preeclampsia was defined on the basis of the presence of one of the following symptoms or signs in the presence of preeclampsia i.e. blood pressure ≥ than 160/110 mmHg (readings were taken on two events at least 6 h apart), proteinuria ≥5 g in 24 h urine specimen (or ≥ 3+ on two urine samples at least 4 h apart), oliguria (urine volume < 500 ml / 24 h), cerebral or visual disturbances, pulmonary edema or cyanosis, epigastric or right upper quadrant pain, platelet count less than 100,000/mm^3^, presence of haemolysis, elevated liver enzymes and low platelets (HELLP) syndrome and fetal growth restriction. Eclampsia was defined as the occurrence of convulsions in women with preeclampsia [17]. Early and late onset preeclampsia was defined as the development of preeclampsia before 34 weeks and at or after 34 weeks of gestation, respectively [18].

Inclusion criteria for controls were pregnant women greater than 20 weeks of gestation in the absence of diagnostic criteria for preeclampsia until discharge of pregnant women after delivery of the baby. Subjects with the history of chronic hypertension, renal diseases, multiple pregnancy, molar pregnancy, diabetes mellitus, chronic infectious diseases, thromboembolic events, and antiphospholipid syndrome were excluded from the study. Controls recruited were matched for age, ethnicity and parity.

### SNVs selection and sample size

Six SNVs *F5*:c.1601G > A(g.chr1:169549811), *F5*:c.6665A > G(g.chr1:169514323;rs6027), *MTHFR*:c.665C > T(g.chr1:11796321), *MTHFR*:c.1286A > C(g.chr1:11794419), *VEGFA*:c.-2055A > C (g.chr6:43768652) and *VEGFA*:c.*237C > T (g.chr6:43784799) were included in the current study. The selection of the SNVs was based on the role of the respective gene in the placenta and the association with preeclampsia in other world populations [3, 19, 20]. Secondly, there are very few published data on the genetic role of SNVs in preeclamptic Pakistani women [16] and the selected SNVs have not been studied in association with preeclampsia in Pakistani patients.

The sample size was calculated by taking the prevalence of combined thrombophilic mutations (*F5*:c.1601G > A and *MTHFR*:c.665C > T) as described by Mello G et al. [21]. Assuming the prevalence as 19.8% in cases and 5.3% in controls, the sample size was calculated at 95% confidence level with alpha = 0.05 and > 80% power, using Ausvet Epitools Epidemiological calculator (http://epitools.ausvet.com.au/). The minimum sample size was calculated to be *n* = 95 in each group, however, to increase the confidence, 125 subjects were selected in each group.

### Sample collection and DNA extraction

Ten ml of venous blood was collected in 50 ml tube containing 400 μl of anticoagulant ethylene diamine tetra acetic acid (EDTA), 0.5 M from both preeclamptic and normal pregnant women. Genomic DNA was extracted by inorganic method, described as previously [22].

### Development of tetra- primer ARMS PCR assay

**Fig. 1:**
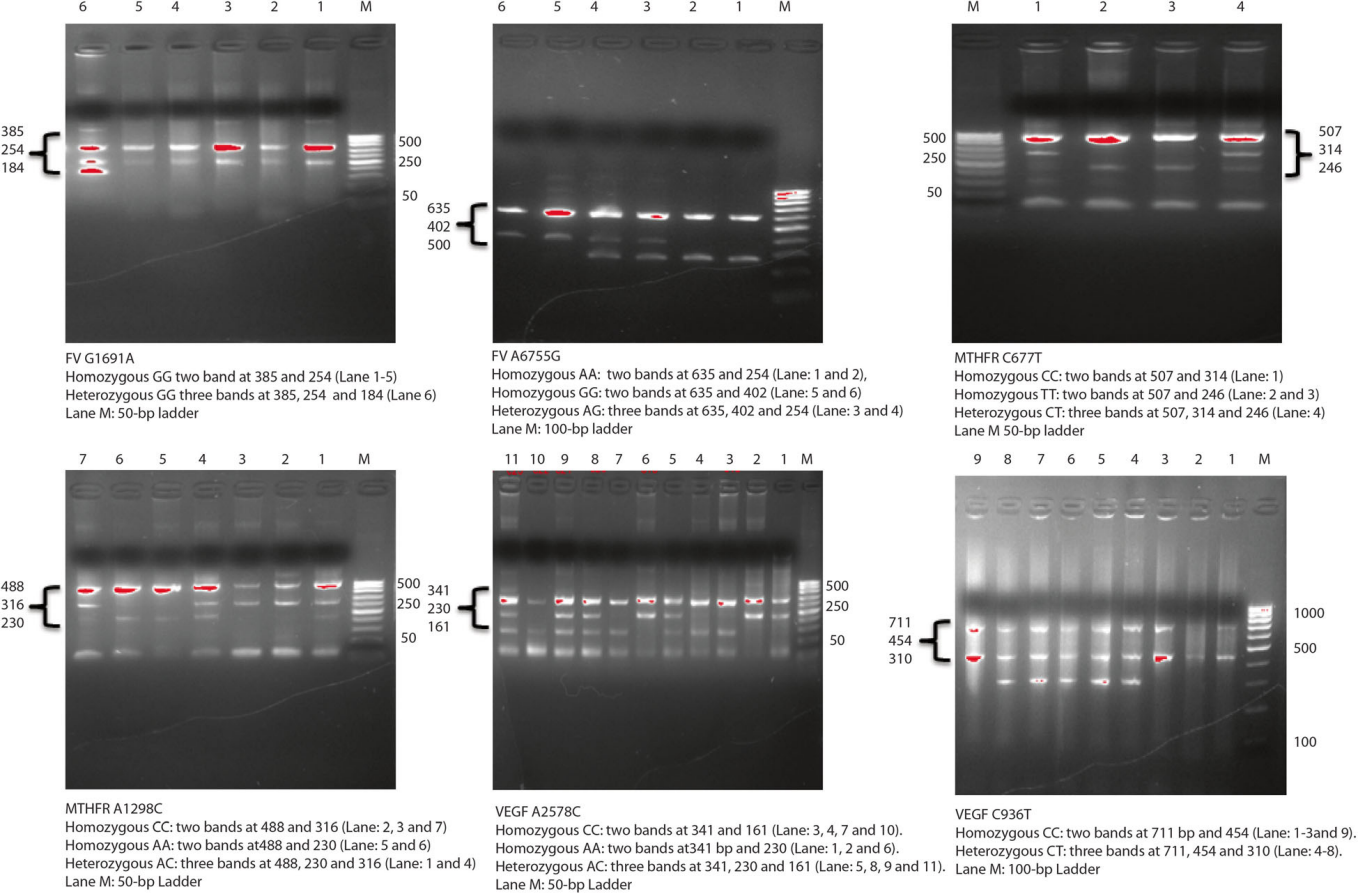
The Agrose gel electrophoresis of newly developed tetra-primer ARMS PCR assays for *F5*, *MTHFR* and *VEGFA* genes variants

Genotyping of variants of selected genes was carried out by tetra-primer amplification refractory mutation system polymerase chain reaction (ARMS PCR) [23]. Primers were designed using PRIMER1 web tool [24]. Primer sequences were confirmed by UCSC In-Silico PCR and Blat-UCSC genome browser websites [25]. Amplification conditions were optimized and desired fragments amplified using 2720 thermocycler (Applied Biosystems). For SNVs *F5*:c.1601G > A and *VEGFA*: c.*237C > T, PCR performed in 20 μl reaction containing 100 ng genomic DNA, 1.25 mM dNTP, 0.6 U Taq polymerase, 2.5 mM MgCl_2_ buffer and10μM of each forward and reverse outer primers and forward and reverse inner primers. PCR reaction for remaining SNVs was carried out in 20 μl reaction containing 100 ng of genomic DNA, 1.25 μM of dNTP, 0.6 U Taq polymerase, 2 mM MgCl_2_ buffer and 8 μM of each forward and reverse outer primers and forward and reverse inner primers. PCR conditions were 95 °C for 5 min, followed by 30 cycles at 94 °C for 30 s, annealing for 45 s, extension at 65 °C for 2 min and a final extension at 72 °C for 10 min. PCR products were separated on a 2% agarose gel (Fig. 1). The selected samples were Sanger sequenced for confirmation of the amplicon and to validate the ARMS assays. Forward and reverse outer primers were used to amplify desired segment for DNA sequencing. Sequences of primers for ARMS assay and product sizes are mentioned in (Table 1).

**Table 1:** Primers sequences, product size and annealing temperatures

SNV/rs	Primers	Sequence	Product size	Annealing
*F5*:c.1601G > A (rs6025)	FI primer (A allele): RI primer (G allele): FO primer (5′ - 3′): RO primer (5′ - 3′):	TAAGAGCAGATCCCTGGACAGTCA ACTTCAAGGACAAAATACCTGTATTCATC TTTGAATATATTTTCTTTCAGGCAGGAA AAATGTTATCACACTGGTGCTAAAAAGG	Product size for A allele: 184 Product size for G allele: 254 Product size of two outer primers: 385	62 °C
*F5*:c.6665A > G (rs6027)	FI primer (A allele): RI primer (G allele): FO primer (5′ - 3′): RO primer (5′ - 3′):	TCGCCTGGAACTCTTTGGCTGGGA CAGGGGTTTTTGAATGTTCAATTCTAGTAAAGAC GGATAAAAAATTTTCTGGGTTGGGCATGAT AAGCCCTTTTCTTGTTGGTTCTTGATATTTTCC	Product size for A allele: 291 Product size for G allele: 402 Product size of two outer primers: 635	60 °C
*MTHFR*:c.665C > T (rs1801133)	FI primer (T allele): RI primer (C allele): FO primer (5′ - 3′): RO primer (5′ - 3′):	AAGGAGAAGGTGTCTGCGGGCGT CAAAGAAAAGCTGCGTGATGATGAAATAGG CTGAGAGTCATCTCTGGGGTCAGAAGCA GAAGAACTCAGCGAACTCAGCACTCCAC	Product size for T allele: 246 Product size for C allele: 314 Product size of two outer primers: 507	60 °C
*MTHFR*:c.1286A > C (rs1801131)	FI primer (A allele): RI primer (C allele): FO primer (5′ - 3′): RO primer (5′ - 3′):	TGTGGGGGGAGGAGCTGACCAGTGAGGA GGTAAAGAACGAAGACTTCAAAGACACCTG GCAAATACATCTTTGTTCTTGGGAGCGGG TACCCTTCTCCCTTTGCCATGTCCACAG	Product size for A allele: 230 Product size for C allele: 316 Product size of two outer primers: 488	60 °C
*VEGFA*: c.-2055A > C (rs699947)	FI primer (C allele): RI primer (A allele): FO primer (5′ - 3′): RO primer (5′ - 3′):	AGCTGTAGGCCAGACCCTGGTAC AGTCAGTCTGATTATCCACCCAGACCT CTTAGGACACCATACCGATGGAACTG GCATATAGGAAGCAGCTTGGAAAAATTC	Product size for C allele: 161 Product size for A allele: 230 Product size of two outer primers: 341	60 °C
*VEGFA*:c.*237C > T (rs3025039)	FI primer (T allele): RI primer (C allele): FO primer (5′ - 3′): RO primer (5′ - 3′):	ATTCCCGGGCGGGTGACCCAGAAT AATGGCGAATCCAATTCCAAGAGGGAACG ATACTGGGGCTTTCTGCCCCAGGACCAC TGAGTGGGAACATTCCCCTCCCAACTCA	Product size for T allele: 310 Product size for C allele: 458 Product size of two outer primers: 715	62 °C

*FI* forward inner, *RI* reverse inner, *FO* forward outer, *RO* reverse outer

### Statistical analysis

Student’s t-test, two-sided Fisher exact test/Chi-square test were applied to the continuous and categorical variables, respectively. A *p*-value of ≤0.05 was considered as statistically significant. Genotype, allele and haplotype frequencies and Hardy-Weinberg equilibrium (HWE) were calculated. The SNVs association between cases and controls in codominant, dominant, recessive, overdominant and log-additive genetic models, and haplotype association test were performed by logistic regression by using SNPStat software [26]. Odds ratio (OR) and 95% confidence interval were determined to find the association between allelic frequencies between the two groups. To overcome the adjustment of multiple comparisons; false discovery rate (FDR), which is the expected proportion of type 1 errors among all positive tests, controlled by applying the step-up approach of Benjamini and Hochberg was used [27]. For the calculation, the FDR online calculator was used (https://tools.carbocation.com/FDR). To assess the genetic association of onset and severity of preeclampsia with SNVs that were found significant; odds ratios were estimated by multinomial logistic regression model by using SPSS version 20.

## Results

### Demographic and clinical characteristics

The demographic and clinical characteristics of the participants are shown in Table 2.

**Table 2:** Demographic and clinical characteristics of participants

Variables		Cases	Controls	*p*-value
Age at presentation (years)		26.7 ± 5	26.6 ± 4	0.972
Age at marriage (years)		21.6 ± 4	21.1 ± 4	0.615
Height (cms)		152.4 ± 8	152.5 ± 8	0.610
Weight (Kg)		54.1 ± 9	54.8 ± 10	0.853
BMI (Kg/m^2^)		23.3 ± 4	22.9 ± 3	0.882
SBP (mmHg)		160.4 ± 20	114.7 ± 8	**< 0.001**
DBP (mmHg)		107 ± 17	78.7 ± 7	**< 0.001**
Gestational age *n* (%)	< 34 weeks	50 (40%)	27 (21.6%)	**0.002**
	> 34 weeks	75 (60%)	98 (78.4%)	
Family history *n* (%)	Absent	106 (84.8%)	122 (97.6%)	**< 0.001**
	Present	19 (15.2%)	3 (2.4%)	
Ethnic distribution of the preeclamptics: *n* (%)				
Sindhi: 70 (56%)				
Urdu: 34 (27.2%)				
Balochi: 4 (3.2%)				
Pashto: 3 (2.4%)				
Punjabi: 2 (1.6%)				
Others: 12 (9.6%)				

*%* percentage, *BMI* body mass index, *cms* centimeters, *DBP* diastolic blood pressure, *Kg* kilogram; *m*^*2*^ square meter, *SBP* systolic blood pressure, *mmHg* millimeters of mercury, *n* number. Bold fonts indicate significant *P*-value

Among the demographic variables; age at marriage, height, weight and body mass index (BMI) were not different between preeclamptic and control groups (*P* > 0.05). The gestational age at presentation and family history were found significantly different between both groups. The preeclamptic and control groups were matched for ethnicity; the ethnic distribution of the preeclamptic patients is presented in Table 2. Fifty (40%) patients presented with early onset and 75 (60%) with late onset preeclampsia whereas, 55 (44%) preeclamptic women developed mild preeclampsia and 70 (56%) presented with severe preeclampsia.

### Genotype distributions and allele frequencies

The genotype distribution of the six SNVs in both control and preeclamptic groups were concordant with HWE. The genotype frequencies and association test are presented in Table 3.

**Table 3:** Allele and genotype frequencies of SNVs in preeclamptics and controls

SNV	Genetic model/ HWE (*p*)	Allele/Genotype	Cases *n* (%)	Controls *n* (%)	OR (95%CI)	*p*-value	P*
*F5*:c.1601G > A		G	246 (98)	249 (99.6)	1.00	0.2125	0.35
		A	04 (2)	01 (0.4)	4.05 (0.45–36.48)		
	N/A	G/G	121 (96.8)	124 (99.2)	1.00	0.16	0.28
		G/A	4 (3.2)	1 (0.8)	4.10 (0.45–37.16)		
	HWE (*p*)	–	0.85	0.96	–	–	
*F5*:c.6665A > G		A	232 (93)	219 (88)	1.00	0.0532	0.099
		G	18 (7)	31 (12)	0.55 (0.30–1.00)		
	Codominant	A/A	109 (87.2)	95 (76)	1.00	**0.036**	0.077
		A/G	14 (11.2)	29 (23.2)	0.42 (0.21–0.84)		
		G/G	2 (1.6)	1 (0.8)	1.74 (0.16–19.53)		
	Dominant	A/A	109 (87.2)	95 (76)	1.00	**0.021**	0.053
		A/G-G/G	16 (12.8)	30 (24)	0.46 (0.24–0.90)		
	Recessive	A/A-A/G	123 (98.4)	124 (99.2)	1.00	0.56	0.71
		G/G	2 (1.6)	1 (0.8)	2.02 (0.18–22.53)		
	Overdominant	A/A-G/G	111 (88.8)	96 (76.8)	1.00	**0.011**	**0.038**
		A/G	14 (11.2)	29 (23.2)	**0.42 (0.21–0.84)**		
	Log- additive	–	–	–	0.55 (0.30–1.02)	0.052	0.104
	HWE (*p*)	–	0.12	0.69	–	–	
*MTHFR*:c.665C > T		C	224 (90)	240 (96)	1.00	**0.0076**	**0.0425**
		T	26 (10)	10 (4)	**2.78 (1.31–5.90)**		
	Codominant	C/C	102 (81.6)	116 (92.8)	1.00	**0.026**	0.06
		C/T	20 (16)	8 (6.4)	2.84 (1.20–6.73)		
		T/T	3 (2.4)	1 (0.8)	3.41 (0.35–33.32)		
	Dominant	C/C	102 (81.6)	116 (92.8)	1.00	**0.0071**	**0.0497**
		C/T-T/T	23 (18.4)	9 (7.2)	**2.91 (1.29–6.57)**		
	Recessive	C/C-C/T	122 (97.6)	124 (99.2)	1.00	0.3	0.42
		T/T	3 (2.4)	1 (0.8)	3.05 (0.31–29.72)		
	Overdominant	C/C-T/T	105 (84)	117 (93.6)	1.00	**0.015**	**0.046**
		C/T	20 (16)	8 (6.4)	**2.79 (1.18–6.59)**		
	Log- additive	–	–	–	**2.48 (1.20–5.13)**	**0.0085**	**0.0396**
	HWE (*p*)	–	0.12	0.17	**–**	**–**	
*MTHFR*:c.1286A > C		A	168 (67)	132 (53)	1.00	**0.0011**	**0.0308**
		C	82 (33)	118 (47)	**0.55 (0.38–0.78)**		
	Codominant	A/A	61 (48.8)	40 (32)	1.00	**0.0098**	**0.0392**
		A/C	46 (36.8)	52 (41.6)	0.58 (0.33–1.02)		
		C/C	18 (14.4)	33 (26.4)	**0.36 (0.18–0.72)**		
	Dominant	A/A	61 (48.8)	40 (32)	1.00	**0.0066**	0.0616
		A/C-C/C	64 (51.2)	85 (68)	0.49 (0.30–0.83)		
	Recessive	A/A-A/C	107 (85.6)	92 (73.6)	1.00	**0.018**	0.0503
		C/C	18 (14.4)	33 (26.4)	0.47 (0.25–0.89)		
	Overdominant	A/A-C/C	79 (63.2)	73 (58.4)	1.00	0.44	0.58
		A/C	46 (36.8)	52 (41.6)	0.82 (0.49–1.36)		
	Log -additive	–	–	–	**0.60 (0.42–0.84)**	**0.0024**	**0.0336**
	HWE (*p*)	–	0.069	0.073	**–**	**–**	
*VEGFA*: c.-2055A > C		C	138 (55)	135 (54)	1.00	0.7876	0.8821
		A	112 (45)	115 (46)	0.95 (0.67–1.35)		
	Codominant	C/C	36 (28.8)	35 (28)	1.00	0.95	0.95
		A/C	66 (52.8)	65 (52)	0.99 (0.55–1.76)		
		A/A	23 (18.4)	25 (20)	0.89 (0.43–1.86)		
	Dominant	C/C	36 (28.8)	35 (28)	1.00	0.89	0.958
		A/C-A/A	89 (71.2)	90 (72)	0.96 (0.55–1.67)		
	Recessive	C/C-A/C	102 (81.6)	100 (80)	1.00	0.75	0.91
		A/A	23 (18.4)	25 (20)	0.90 (0.48–1.69)		
	Overdominant	C/C-A/A	59 (47.2)	60 (48)	1.00	0.9	0.93
		A/C	66 (52.8)	65 (52)	1.03 (0.63–1.70)		
	Log- additive	–	–	–	0.95 (0.66–1.37)	0.78	0.91
	HWE (*p*)	–	0.59	0.72	–	–	
*VEGFA*:c.*237C > T		C	229 (92)	222 (89)	1.00	0.2939	0.433
		T	21 (8)	28 (11)	0.73 (0.40–1.32)		
	N/A	C/C	104 (83.2)	97 (77.6)	1.00	0.26	0.404
		C/T	21 (16.8)	28 (22.4)	0.70 (0.37–1.31)		
	HWE (*p*)	–	0.305	0.36	–	–	

*HWE* hardy-weinberg equilibrium; P*: Benjamini-Hochberg adjusted *P* value. Bold fonts indicate significant *P*-value

The *F5*:c.6665A > G variant showed 0.42 fold decreased risk for preeclampsia (A/G vs AA/GG: 95% CI = 0.21–0.84; P* = 0.038) in the overdominant model. It was also found to be associated with preeclampsia in the codominant and dominant models; however, the association lost after FDR correction.

Furthermore, significantly increased risk for preeclampsia was found with *MTHFR*: c.665C > T, in dominant (CT/TT versus CC: OR = 2.91, 95% CI = 1.29–6.57; P* = 0.0497), overdominant (CT versus CC/TT: OR = 2.79, 95% CI = 1.18–6.59; P* = 0.046) and log-additive (OR = 2.48, 95% CI = 1.20–5.13; P* = 0.0396) models.

The significantly decreased risk was associated with CC genotype of *MTHFR*: c.1286A > C variant in codominant (CC versus AA: OR = 0.36, 95% CI = 0.18–0.72; P* = 0.0392) and log-additive (OR = 0.60, 95% CI = 0.42–0.84; P* = 0.0336) models whereas association in dominant and recessive models did not remain significant after applying FDR. We did not find a significant association of *VEGFA*: c.-2055A > C, *VEGFA*: c.*237C > T and *F5*:c.1601G > A with preeclampsia in Pakistani women.

Allele frequencies for *MTHFR*: c.665C > T and c.1286A > C were significantly different among cases and control groups (P* = 0.0425; P* = 0.0308, respectively). The frequencies of preeclamptics according to the onset and severity of preeclampsia were further compared with the genotype distribution of *F5*:c.6665A > G and *MTHFR* variants (Table 4).

**Table 4:** Association of *F5*:c.6665A > G, *MTHFR*: c.665C > T and c.1286A > C genotypes with onset and severity of preeclampsia

SNV	Genotype	Onset of preeclampsia					Severity of preeclampsia				
		Early (*N* = 50)	Late (*N* = 75)	OR (95% CI)	*p*-value	P*	Mild (*N* = 55)	Severe (*N* = 70)	OR (95% CI)	*p*-value	P*
*F5*:c.6665A > G	AA	43 (34.4%)	66 (52.8%)	1.00	–	–	50 (40%)	59 (47.2%)	1.00	–	–
	AG	6 (4.8%)	8 (6.4%)	1.27 (0.41–3.40)	0.68	1.02	4 (3.2%)	10 (8%)	0.46 (0.13–1.56)	0.21	0.84
	GG	1 (0.8%)	1 (0.8%)	1.51 (0.09–25.51)	0.77	1.026	1 (0.8%)	1 (0.8%)	1.12 (0.07–18.81)	0.94	0.94
*MTHFR*:c.665C > T	CC	40 (32%)	62 (49.6%)	1.00	–	–	46 (36.8%)	56 (44.8%)	1.00	–	–
	CT	8 (6.4%)	12 (9.6%)	1.08 (0.40–2.88)	0.89	1.068	7 (5.6%)	13 (10.4%)	0.65 (0.24–1.77)	0.40	0.96
	TT	2 (1.6%)	1 (0.8%)	2.88 (0.25–33.20)	0.40	0.8	2 (1.6%)	1 (0.8%)	2.20 (0.19–25.5)	0.53	0.908
*MTHFR*:c.1286A > C	AA	32 (25.6%)	29 (23.2%)	1.00	–	–	27 (21.6%)	34 (27.2%)	1.00	–	–
	AC	8 (6.4%)	38 (30.4%)	**0.19 (0.08–0.49)**	**< 0.001**	**0.012**	15 (12%)	31 (24.8%)	0.65 (0.28–1.5)	0.31	0.92
	CC	10 (8%)	8 (6.4%)	1.08 (0.37–3.19)	0.89	0.97	13 (10.4%)	5 (4%)	3.26 (1.03–10.30)	**0.04**	0.24

P*: Benjamini-Hochberg adjusted *P* value. Bold fonts indicate significant *P*-value

It was observed that the frequency of heterozygous *MTHFR*:c.1286A > C genotype was different between preeclamptics presented at less than 34 weeks of gestation as compared to patients presented at or after 34 weeks. Preeclamptics with *MTHFR:* c.1286A > C*,* AC genotype were found to be 0.19 times less susceptible to present with early onset preeclampsia (95% CI = 0.08–0.49; P* = 0.012) as compared to late onset. However; *MTHFR*: c.1286A > C, association with severity of preeclampsia did not remain significant at Benjamini-Hochberg adjusted *P*-value*.* The haplotype frequencies of variants in preeclamptic and controls; and linkage disequilibrium are shown in Table 5.

**Table 5:** Haplotype frequencies of *F5, MTHFR* and *VEGFA* gene variants and linkage disequilibrium analysis

Haplotype	*F5*: c.1601G > A	*F5*: c.6665A > G	Cases	Control	Total frequency	OR (95%CI)	*p*-value	P*
1.	G	A	0.928	0.876	0.902	1.00	–	–
2.	G	G	0.056	0.120	0.088	**0.35 (0.17–0.72)**	**0.0048**	**0.0144**
3.	A	G	0.016	0.004	0.01	7.05 (0.72–69.36)	0.095	0.142
4.	A	A	–	–	–	–	–	–
Global haplotype association *p*-value: 0.0045								
Linkage disequilibrium analysis: D’ = 0.991, r2 = 0.091; *p*-value < 0.001								
Haplotype	*MTHFR*: c.665C > T	*MTHFR*: c.1286A > C						
1.	C	A	0.672	0.528	0.6	1.00	–	–
2.	C	C	0.224	0.432	0.328	**0.32 (0.21–0.51)**	**< 0.0001**	**0.0006**
3.	T	C	0.104	0.04	0.072	**2.85 (1.29–6.29)**	**0.0099**	**0.0198**
4.	T	A	–	–	–	–	–	–
Global haplotype association *p*-value**<** 0.0001								
Linkage disequilibrium analysis: D’ = 0.999, r^2^ = 0.116; *p*-value < 0.001								
Haplotype	*VEGFA*: c.*237C > T	*VEGFA*: c.-2055A > C						
1.	C	C	0.552	0.54	0.546	1.00	–	–
2.	C	A	0.364	0.348	0.356	1.26 (0.73–2.17)	0.41	0.41
3.	T	A	0.084	0.112	0.098	0.66 (0.34–1.25)	0.20	0.24
4.	T	C	–	–	–	–	–	
Global haplotype association *p*-value: 0.38								
Linkage disequilibrium analysis: D’ = 0.9987, r^2^ = 0.13; *p*-value < 0.001, Bold fonts indicate significant *P*-value								

The G-G haplotype of *F5*:c.1601G > A and c.6665A > GG, and C-C and T-C haplotypes of *MTHFR*: c.665C > T and c.1286A > C variants showed significant association with preeclampsia.

## Discussion

Preeclampsia is a complex disorder involving the role of multiple genes related to placental pathophysiology. Variations of several genes have been studied in preeclamptic patients belonging to different populations and ethnic groups [6]. Pakistani population is genetically heterogeneous and has remained an excellent source to study the relationship between gene and disease. Despite numerous genetic studies conducted in different populations [3], to the best of our knowledge, only one study reported the association of angiotensin-converting enzyme (*ACE*) gene I/D variant with preeclampsia in Pakistani population [16]. Preeclampsia has been related to thrombophilia and the candidate genes commonly involve *F5*:c.1601G > A, *MTHFR*: c.665C > T and c.1286A > C variants. Although various studies have reported an association of SNVs with preeclampsia [19, 28], the role of *F5*, *MTHFR* and *VEGFA* gene variants in Pakistani preeclamptic women have not been determined yet. Our data showed significant differences in the genotype frequencies of *MTHFR*: c.665C > T, c.1286A > C and *F5*:c.6665A > G variants in different genetic models and allelic frequencies of *MTHFR*: c.665C > T, and c.1286A > C variants between preeclamptic women and controls.

This is the first study to report an association of *F5*:c.6665A > G variant with preeclampsia in any population. A previous study investigated the role of 20 missense variations of the *F5* gene in Japanese preeclamptic women, including *F5*:c.6665A > G and indicated the significant association of only two variants rs6033 and rs6020. *F5*:c.6665A > G variant was not found significant in association with preeclampsia in Japanese patients [10], which is in disagreement with our findings revealing decrease risk to preeclampsia. *F5*:c.6665A > G results in the substitution of aspartic acid into glycine in the C2 terminal domain of the *F5* gene. The mutated residue is smaller, neutral and more hydrophobic than the wild type [29]. The substitution may affect the hydrogen bond formation, ionic interaction and rigidity of the protein. It may have an influence on the conformational changes in the protein and may be associated with unexplained activated protein C (APC) resistance [9–11]. *F5*:c.6665A > G has been reported as the likely benign functional variant and have significantly higher frequency among Asians and Arabians [30].

We did not find any association of *F5*:c.1601G > A with preeclampsia in our cohort. Though the *F5* is one of the thrombophilic genes and its variants may have a key role in the development of the preeclampsia; however, the association of *F5*:c.1601G > A with thrombophilia and preeclampsia has remained controversial among studies; conducted on patients with different ethnic groups [31]. It has been found that *F5*:c.1601G > A is common in Northern India and predisposes women to preeclampsia [20]; which is contradictory to our results, though patients may have common ethnic lineages. In agreement with our study, no significant relationship of *F5*:c.1601G > A with preeclampsia was found in Turkish and Iranian patients [32, 33]. This finding strengthens the hypothesis that *F5* gene variations may have a role in predisposition to preeclampsia, but the type of variants may vary among different populations and geographical locations.

The results of the present study exhibited the increased risk of preeclampsia with CT genotype under the overdominant model, and with the T allele of *MTHFR*: c.665C > T. In a meta-analysis, Yang et al. [34] included 57 different studies that showed TT as a risk factor for preeclampsia in Caucasians, South Americans, East Asians and Africans whereas, TT + CT was observed as a risk factor in East Asians. In the same study, a protective role was observed with CC, CT, and CC + CT in East Asians and CT in South Asians; however, no significant association was found in Hispanics and Middle East population. Another meta-analysis demonstrated a 1.45-fold increased risk for preeclampsia with CT genotype in East Asians [35]. Contrary to these findings and the present study, Aggarwal et al. [20] found T allele protective against preeclampsia in North Indian women.

*MTHFR*: c.665C > T is a missense variant, resulting in the substitution of alanine to valine. This leads to the production of thermolabile protein product which possesses reduced catalytic activity. The TT and CT genotypes have shown 20–65% of reduced enzyme activity to process folic acid as compared to CC genotype. Further, this variant increases the risk of hyperhomocysteinemia aggravated with folic acid deficiency [36, 37]. PolyPhen prediction indicated it as probably damaging, whereas HOPE inference showed that affected residue is bigger than wild type and located in a domain that is important for the protein activity and its interactions with other domains [29, 38]. *MTHFR*:c.1286A > C has been associated with reduced enzyme activity, though not with thermolability [36]. PolyPhen predicted it to be benign. HOPE analysis predicted the affected residue as neutral and more hydrophobic that might disturb correct folding [29, 38].

In addition, we also found the significant association of *MTHFR*: c.1286A > C with preeclampsia. This is contradictory with the results reported in Australian [39], Dutch [40] and Mexican [41] women; where the studies have reported lack of association of *MTHFR* gene variants with preeclampsia which may be due to genetic heterogeneity and different ethnic backgrounds of the patients. However, the study from Southeast of Iran reported an association of *MTHFR*:c.1286A > C with preeclampsia suggesting its role as a risk factor for preeclampsia in Asians; though in contrast to the findings of a current study they observed AC genotype as a risk factor for preeclampsia [19].

We analysed the association of *F5*:c.6665A > G and *MTHFR* variants between early and late onset preeclampsia. Previously, *MTHFR*:c.1286A > C, AC and CC genotypes and *F5*:c.1601G > A, GA genotype have been associated with over 2.5-fold increased risk for early onset preeclampsia [19]. Contradictory to these findings, our study suggested less risk for early onset preeclampsia with the AC genotype of *MTHFR*: c.1286A > C. This suggests further research to explore the role of genotypes in severity and outcome.

VEGF is expressed in the placenta and has a significant role in its development and maintenance. *VEGFA* variants have been widely studied and its association with preeclampsia has been found in several studies, including Chinese, Brazilian, Hungarian and Korean patients [42–45]. However, our study negates the association of *VEGFA*: c.-2055A > C and c.*237C > T variants with preeclampsia in Pakistani patients. There is no significant difference found among allelic frequencies between cases and controls. These findings are in agreement with the studies of *VEGFA* variants, comprising of North American, Greece, Mexican and Sri Lankan patients [46–51]. The differences in results in various studies may be due to different inclusion criteria for cases and controls, sample sizes, different geographical locations, environmental factors as well as different ethnicities and genetic features.

The Pakistani population consists of at least 18 ethnic groups with more than 60 spoken languages [52]. The major ethnicities consist of Punjabi, Sindhi, Balochi, Urdu speaking, Pathan and Saraiki groups. All the ethnic groups have different genetic lineages resulting in genetic heterogeneity. Punjabi ethnics have a complex admixture of South Asian, East Asian and West Eurasian lineages, whereas, Pathan, Balochi and Sindhi share alleles with Greeks and Georgians [53–55]. While the Urdu speaking ethnic community has heterogeneous Indian ancestry [56]. Thus the genetic variations exhibit significant differences in the risk of developing various disorders and disease progression in the Pakistani population [15, 57, 58].. Majority of the preeclamptic patients in our study were Sindhi (56%) and Urdu (27.2%) speaking. The frequencies and genotypes of the genes analysed in this study, support the ethnic biases of the genetic variants. The frequency of the *MTHFR*: c.665C > T allele is up to 16.7% in Indian ethnic groups with the highest frequency of 7.8% of the TT genotype in the Rajput ethnicity. Whereas, *MTHFR: c.1286A > C* allele was found higher among Dravidians of east India and south India [59]. Moreover, *F5*:c.1601G > A heterozygous are more frequent in European descendants and carry 5 to 9% of the *F5*:c.1601G > A heterozygotes, as compared to less than 1% among Asians and African descendants [60].

In the present study, non-significant differences in BMI between preeclamptics and control groups were observed. There may be several factors which may affect the BMI, such as differences among residents of urban and rural areas, low and middle-income countries and the socioeconomic status of participants [61–63]. In the study, majority of the preeclamptics were referred from the rural areas of Sindh and had lower socioeconomic status; there was non-availability of pre-pregnancy BMI records and lack of knowledge regarding self-measurement. BMI in the present study was recorded at the time of presentation in both groups. The current findings are supported by a multicentre study conducted in Pakistan that did not find a significant association of BMI with preeclampsia [64]; furthermore a meta-analysis conclusively reported BMI as a weak predictor for the preeclampsia [65].

There are certain limitations in the present study. Though our sample population included some major ethnicities of Pakistan including Sindhi and Urdu ethnic groups; ethnicities of other provinces were in minority and for this reason ethnic diversities related to preeclampsia may not be generalized and require large scale studies in other provinces. Similarly, limited literature availability on genetic aspects in Pakistani preeclamptic women presented obstacles in the comparison of present study findings. We only investigated three genes, so it is possible that other genes may have a role in the development of preeclampsia in the Pakistani population.

## Conclusion

*MTHFR*: c.665C > T variant was associated with preeclampsia, whereas *F5*:c.6665A > G and *MTHFR*: c.1286A > C variants may have a protective effect against preeclampsia in Pakistani pregnant women. The significant association of SNVs for predisposition to preeclampsia may require further research for identification of more genetic variants related to preeclampsia genes. This may help to better understand the pathophysiological mechanisms of preeclampsia and may pave paths for effective therapeutic approaches. Furthermore, the development of cost effective ARMS assays may be a rapid, simple and economical method to genotype the SNVs for further studies.

## Data Availability

The data of the manuscript will be provided on the request, by the corresponding author.

## Abbreviations

*ACE*: Angiotensin-converting enzyme
APC: Activated protein C
ARMS: Amplification refractory mutation system
BMI: Body mass index
CI: Confidence interval
EDTA: Ethylene diamine tetra acetic acid
*F5*: Factor 5
FDR: False discovery rate
HELLP: Haemolysis elevated liver enzymes and low platelets
HWE: Hardy-Weinberg equilibrium
*MTHFR*: Methylenetetrahydrofolate reductase
OR: Odds ratio
PCR: Polymerase chain reaction
SNVs: Single nucleotide variants
*VEGFA*: Vascular endothelial growth factor A
